# Development and evaluation of the Good Grief program for young people bereaved by familial cancer

**DOI:** 10.1186/s12904-021-00752-z

**Published:** 2021-04-29

**Authors:** Pandora Patterson, Fiona E. J. McDonald, Elizabeth Kelly-Dalgety, Bianca Lavorgna, Barbara L. Jones, Anna E. Sidis, Thomasin Powell

**Affiliations:** 1 Canteen Australia, GPO Box 3821, Sydney, NSW 2001 Australia; 2 Faculty of Medicine and Health, The University of Sydney, Sydney, Australia; 3 Present address: Support After Suicide, Jesuit Social Services, Australia and the Faculty of Education, Monash University, Melbourne, Australia; 4 Steve Hicks School of Social Work and Dell Medical School, The University of Texas at Austin, Austin, USA; 5 Present address: School of Psychology, University of Wollongong, Wollongong, Australia

**Keywords:** bereavement, adolescent, young adult, program development, outcomes, parental cancer, sibling cancer

## Abstract

**Background:**

Adolescents and young adults (AYAs) bereaved by the death of a parent or sibling from cancer report unique psychosocial needs and can have difficulty adjusting to their loss. Unaddressed, this can result in poor long-term bereavement outcomes. This paper describes the development and evaluation of Good Grief *–* a 3-day camp-based program focused on meeting coping, social support, and respite needs of AYAs bereaved by familial cancer.

**Methods:**

One hundred and nine Australian AYAs (68% female; age: 12–25 years, *M* = 16.63) participated in the evaluation. Grief intensity (Texas Revised Inventory of Grief), meaning-making (Grief and Meaning Reconstruction Inventory), trauma coping (Perceived Ability to Cope with Trauma Scale) and unmet needs (Bereaved Cancer Needs Instrument) measures were administered pre-program and 3-months post-program. Acceptability was measured after each session and at the program’s conclusion. Appropriateness was measured at 3-month follow-up. Thirteen participants were interviewed three months post-program on their perceptions of the program.

**Results:**

Participants reported high program satisfaction, engagement with psychosocial sessions, and enjoyment of recreational activities. Significant improvements were observed in trauma coping abilities and reductions in unmet needs for managing emotions, social support, respite, future planning, and accessing information and support domains. No change was evident in grief intensity or meaning-making as measured quantitatively. Interviews supported these quantitative findings but also identified evidence of personal growth, a component of meaning-making.

**Conclusions:**

Good Grief is a highly acceptable and beneficial intervention that addresses the unique needs of AYAs bereaved by familial cancer.

## Background

### The impact of bereavement on AYAs

The death of a parent or sibling can be a distressing and traumatic event at any life stage. However, during adolescence and young adulthood (AYA; approximately 12–25 years of age) – a time characterised by emotional, psychological and physical development and identity formation – such a loss can be particularly stressful and impactful, having short- and long-term effects on wellbeing [1, 2]. AYAs who are bereaved of a parent or sibling can experience decline in academic performance, school abandonment, feelings of anger, loneliness, isolation, and symptoms of anxiety and depression following their loss [3–7]. Bereaved sibling AYAs also commonly report sleep disturbance [6], and parentally bereaved AYAs are more likely to experience separation anxiety, conduct problems, and substance abuse, and show increased functional impairment relative to non-bereaved AYAs [8]. A parent or sibling’s death also often results in numerous practical impacts, including changes to family structure, financial burden, physical and emotional caregiver unavailability, and changes to future plans due to taking on caregiver or income earner responsibilities [7, 9, 10].

### AYAs and cancer bereavement

Regarding cancer bereavement specifically, a systematic review of the child and AYA sibling and offspring literature in this area was recently conducted by Hoffmann, Kaiser, and Kersting [11]. They found that adolescents in particular tend to have a greater vulnerability to behavioural problems and depression/anxiety symptoms in comparison to children and young adults. This is potentially due to adolescents having a greater depth of understanding of the consequences and complexities of death and loss than children [11], but having poorer social and emotional skills to cope with the loss than young adults [12]. Furthermore, Hoffmann et al. [11] reported unresolved grief in approximately 50% of cancer bereaved AYAs two to nine years after the death, and heightened risk of self-injury in young adults who had a parent die of cancer during their teenage years compared with their nonbereaved peers. The review also found that unmet needs in social support were associated with greater risk of poor bereavement outcomes in AYAs (e.g., severe anxiety, unresolved grief).

Patterson and Rangganadhan [13] identified seven key areas of need amongst AYAs as a result of their parent dying of cancer: support and understanding, help coping with feelings, to talk to people who have had a similar experience, information, to have a break/have fun, space and time to grieve, and help with household responsibilities. Building on this work and research into the needs of AYAs who had experienced the death of their parent or sibling to cancer, the Bereaved Cancer Needs Instrument (BCNI) [14, 15] was developed. The authors found that for both offspring and sibling AYAs, the two domains with the greatest percentage of unmet needs were social support from other bereaved AYAs and respite (having a break from grief/having fun) [14]. These were followed by broader social support, information about grief, and coping with emotions domains. Furthermore, AYAs with more unmet needs reported higher levels of psychological distress.

### Existing AYA interventions

Although there is a clear need for psychosocial support for bereaved AYAs (both those bereaved by cancer or due to other causes) to assist them effectively cope with and adjust to loss, there is a dearth of bereavement interventions for AYAs. Those that do exist are mostly targeted at children and younger AYAs (generally ages 6–17) [16–21] rather than being AYA specific and therefore, particularly for older AYAs, may not adequately address the needs unique to their developmental stage [22]. Additionally, few existing interventions are specific to cancer bereavement. In a recent review by Ing et al. [22], only three of the twenty-two bereavement interventions for young people that were examined were specifically designed for those who had experienced the death of a family member from cancer, and of these, none were specific to AYAs.

Design and measurement issues also exist in the literature. As highlighted in several systematic reviews of the current literature [22–24], many existing bereavement support programs lack the use of theoretical frameworks to drive and guide the design of the program’s intervention and evaluation. Further, programs have tended to focus on normalising grief and providing a space for participants to talk about and process their grief, but do not teach coping strategies for adjusting to the loss [22]. Those that did mainly employed cognitive behavioural therapy techniques, emphasising restructuring of the ‘negative’ emotions that arise with grief and loss [22]. It has been argued that this focus may encourage inflexible avoidance of these normal emotions, potentially inhibiting healthy grieving processes in participants [22, 25–27]. Finally, measurement issues are also evident, including a lack of pre-post and follow-up outcome measures, and reliance on parent-reports and facilitator-reports rather than by participants themselves [22, 23].

To minimise the likelihood of negative short- and long-term consequences, it is essential that evidence-based and age-appropriate support is available to AYAs who have experienced the death of a parent or sibling due to cancer. To address this, we developed Good Grief (GG) – a 3-day, 2-night psychosocial program*.* The GG program was designed to provide a safe and supportive environment for AYAs to achieve the following outcomes: increased coping abilities to better manage grief; reduced unmet information, respite, social support, psychological support, and practical support needs; and increased post-loss meaning-making (sense of personal growth and valuing of life since the death). The name ‘Good Grief’ was chosen for the program in consultation with some of Canteen’s clients, encapsulating the idea that the grief process is a natural, normal and healthy (as opposed to a negative or abnormal) outcome following the loss of a loved one.

### Theoretical and therapeutic frameworks

The following theoretical and therapeutic frameworks were used in developing the GG program and informing its outcomes.

#### The dual process model (DPM)

The DPM posits that within the context of daily life, bereaved individuals engage in coping processes in relation to two kinds of stressor – loss and restoration [28, 29]. Loss-oriented coping refers to an individual’s focus on and engagement with the grief/loss experience (e.g., thinking about the lost loved one, their death, life prior to their passing; tearfulness and longing, memories of time spent with their loved one) whereas restoration-oriented coping refers to an individual’s focus on adjusting to life post-loss (e.g., forming new social roles and relationships, mastery of new tasks previously completed by the deceased person, continuing with daily responsibilities, distraction from or avoidance of grief).

Stroebe and Schut [28] postulate that processing of loss does not occur in phases but is an oscillation between loss-orientation and restoration-orientation, which is key to optimal adjustment. Whilst at times bereaved individuals may be caught up in the grief of their loss, at other times they may seek distraction from this, or may have no choice but to attend to other life stressors such as work.

#### Constructivism and meaning making approaches to grief

The constructivist approach theorises that an individual’s understanding of their identity is formed through “the stories that we construct about ourselves and share with others” [30]. In the context of grief and bereavement, the stories and ideas a person may take up to make sense of life can be disrupted [30, 31]. Constructivist approaches such as narrative therapy [32] consider meaning making and re-storying the connection between the young person and the deceased to be central to the grieving process [33, 34]. By reflecting on the way in which the loved one has had and continues to have an impact on their values and characteristics, the bereaved young person can make some sense of their loss and consider how they wish to engage with life following it. Difficulty engaging in this process has been associated with complicated grief symptoms in young adults [31]. Additionally, in a sample of 1022 recently-bereaved university students, sense-making predicted bereavement adjustment [35]; and adolescents’ meaning-making following a life-changing event (including loss) has been associated with higher wellbeing, controlling for baseline wellbeing [36].

#### Self-compassion

Research pioneered by Neff [37] defines self-compassion as openness to (as opposed to avoidance of) one’s own pain and suffering as a common human experience, offering oneself non-judgmental understanding and kindness. In this way, self-compassion can be thought to have three core components – self-kindness in moments of pain or failure (as opposed to self-criticism), common humanity (seeing one’s experiences as being part of the human experience and condition as a whole), and mindfulness (holding unpleasant thoughts and emotions in a “balanced awareness”) [37]. Research has pointed to self-compassion playing a role in psychological resilience, with a number of studies linking self-compassion with well-being and adaptive coping strategies in AYAs [38–41].

#### Continuing bonds

In the bereavement literature, a continuing bond is defined as “the presence of an ongoing inner relationship with the deceased person by the bereaved individual” [42], and is posited as a normal and core feature of the grieving and grief resolution process [43]. A number of studies report maintenance of ongoing attachment to deceased family members in AYAs [44–46], with Hansen et al. [45] finding that this continued bond assisted with meaning-making and bereavement adjustment.

### The current study

This paper describes the development and evaluation of the Good Grief (GG) program – a manualised camp-based program based on the DPM and aimed at meeting the social support, coping and respite needs of AYAs who have had a parent or sibling die from cancer.

## Method

### The development of Good Grief

An advisory group comprising of clinicians with expertise in working with cancer bereaved AYAs, AYA psycho-oncology researchers, and bereaved young people, guided the program design and development. The dual process model of coping with bereavement (DPM) was selected as the primary guide for the development of the program. With this model in mind, GG was designed to encourage active oscillation between the loss and restoration orientations during the camp; moving between therapeutic grief work and recreation/respite activities throughout the camp. This model was also explicitly discussed over the course of the program to assist participants to understand the grief process in greater depth and normalise their experience. In the development of this program we were particularly considerate of the developmental stage of those attending the camp. Therapeutic activities adopted for this program were based on approaches that attend to the formation of identity and values, and normalise rather than pathologise grief responses. Such approaches include acceptance and commitment therapy (ACT), constructivist therapies such as narrative therapy and Kristen Neff’s concept of self-compassion [37]. We also utilised the concept of continuing bonds, known to be helpful in familial bereavement, to assist us in the development of these activities.

During the program, participants were given the opportunity to share stories about their deceased loved one. A modified version of the Life Imprint [33] (see [47] for a detailed description and evaluation of this session) involved inviting friends or family members to write about the values, characteristics and mannerisms that the young person has inherited, or learned from the deceased, and continues to carry with them. A later session adopted aspects of White’s [32] outsider witness process to support young people to share memories and stories, and to respond to other group members supportively. Both of these activities were focussed on increasing social support and connection between group members.

The self-compassion approach was a core feature of GG’s group discussions, as participants were encouraged to show themselves and others kindness and care as they encountered a range of enjoyable and difficult experiences and emotions during the program. Finally, the principles of continuing bonds were implemented throughout GG as participants were given opportunities to practice maintaining a connection with their loved one and commemorate them. Some examples of this are participants creating memory boxes or artworks to remember their loved one, and the Honouring Ceremony session, in which young people participated in a group ritual honouring their loved ones (e.g., writing a letter to their loved one on dissolvable paper and then releasing it into the ocean).

### Good Grief format

GG is a two-night three-day camp-based program to support AYAs following the death of a parent or sibling from cancer. GG aims to provide a space for young people to: increase their coping abilities in order to better manage their grief, address unmet needs (including information, respite, social support, psychological support and practical needs) that often arise following the death of a loved one due to cancer, and make meaning from their loss. Moreover, GG provides a place for young people to share about the life and legacy of their loved one with their peers – to acknowledge the impact their deceased family member has had on their lives, and to honour, grieve and remember them with peers who are going through a similar experience. GG has six psychosocial sessions (see Table 1) interspersed with recreational activities (e.g. water sports, outdoor challenges, group games). Typically, 20–25 AYAs and 4–6 psychosocially trained facilitators (with tertiary education and experience in social work, psychology or equivalent) attended each camp. Psychosocial sessions were conducted in groups of between 8 and 10 AYAs, led by two facilitators.

**Table 1:** Session content and outcomes

*Session*	*Content*	*Purpose/Outcomes*
Introduction of Loved One *(90mins)*	Young people are given an opportunity to introduce the loved one they have lost to the rest of the group and share their experience of loss.	- Begin exploration of personal narratives about grief experience. - Normalise the experience of grief and build the sense of shared experience amongst the group.
Understanding Grief *(90mins)*	The grief process and uniqueness of individuals’ experiences is explored. The dual process model of grief is discussed. Mindfulness is introduced as a grounding activity. Personal strengths are identified by each participant.	- Increase understanding of and normalise the grief process. - Explore personal narratives about grief experience. - Introduce mindfulness for grounding when distressed. - Assist young people to articulate personal strengths as a resource.
Sharing Memories *(90 mins)*	Young people share a memento/photo of their loved one with the group and create a memory box **OR**^a^ create an artistic representation of their memories of their loved one and share it with the group. A mindfulness-based activity ends the session.	- Facilitate the grieving process and continuing bond with loved one who died. - Support young people in engaging with and communicating memories of loved one. - Develop mindfulness skills.
Honouring Ceremony *(60mins)*	The concept of using rituals to keep a connection with deceased loved ones introduced. Young people participate in a group ritual activity to honour the memories of deceased loved ones. Another mindfulness-based grounding activity is completed.	- Commemorate young people’s loved ones and provide an opportunity for them to continue their bond with their loved one. - Explore ways to live well with grief (balance between keeping connections with the loved one and moving forward). - Develop mindfulness skills.
Life Imprint *(90mins)*	Group members identify five personal values and reflect on the origins of their values. Each member is given a letter written by a family member that comments on personal qualities/values shared by the young person and their loved one. Bracelets are made to symbolise these values. The group discuss how they can live out their values.	- Strengthen continuing bonds young people have with loved ones who have died. - Increase knowledge of personal values. - Explore the impact that the loved one has left on the young person and integrate family’s/friend’s perceptions of this. - Increase understanding of what it means to live a meaningful life.
Coping and Resources *(90mins)*	*Grief Support Kit:* A mindfulness-based grounding activity is completed. The group discusses how they can use different items or places at home to self-soothe using their senses. A personalised grief kit is created by each participant using self-soothing items. The session ends with participants identifying strengths they see in one another. **OR**^a^ *Finding Support In Our Lives:* Young people’s values, knowledge, skills and important supports available to them are explored and expressed through metaphor-based activities. The group brainstorms challenges they might face and generates possible coping strategies. To wrap up, a mindfulness activity is completed.	- Identify and increase awareness of existing internal and external coping resources. - Learn coping and distress tolerance strategies for dealing with difficult emotions and situations. - Facilitate peer support and a sense of shared experience. - Develop mindfulness skills.

^*a*^*Two content options are provided for this session. Facilitators would choose which content was best suited to the needs and maturity of the AYAs attending that particular program*

### Participants

AYAs were eligible to participate in the GG program and its evaluation if they were aged between 12 and 25 years and were bereaved of a parent or sibling due to cancer.

### Procedure

AYAs who were already receiving support from Canteen Australia, a national organization that provides psychosocial support to AYAs (12–25 y/o) impacted by cancer (themselves or in their family), were recruited to the program via multiple methods, including flyers, emails, phone calls and face-to-face conversations. The program was provided at no cost to attendees. Information sheets were sent to participants, informing them of the purpose of the program’s evaluation, and that participation was voluntary and would not affect their ability to attend the program or their relationship with Canteen. Consent/assent was received from all participants and a parent/guardian if the participant was under age 18. Data were collected via paper surveys and interviews. AYAs completed demographics questions and baseline outcome measures at the beginning of the program (T0). Missing demographic data was obtained from Canteen’s client management system. They completed a program satisfaction survey at the conclusion of the program (T1). In the T2 survey, completed three months post-program, participants responded to a measure assessing usefulness, relevance and practicability and outcome measures. AYAs also completed session engagement surveys at the immediate conclusion of each program session. Participants were assigned an ID number which was used to match their surveys at each timepoint (apart from the session satisfaction surveys, which were anonymous). AYAs who consented to be contacted for an interview were contacted after T2, at which point an interview was scheduled if they agreed to take part.

To understand the impact of this program we used aspects of a framework for implementation outcomes [48] to assess the acceptability and appropriateness of the program and examined objective outcome measures. Qualitative semi-structured interviews were conducted with participants to gain deeper understanding of AYAs’ perceptions of the program. Details are provided below.

### Measures

See Table 2 for a description of acceptability, appropriateness, and outcome measures used in surveys to evaluate the program.

**Table 2:** Targeted concepts, names and psychometric features of survey acceptability, appropriateness, and outcome measures

*Concept/variable – measure name*	*No of items/subscales*	*Response scale*	*Example item/s*	*Validity and reliability evidence*
Acceptability: End of session engagement (measuring perceived session helpfulness, meaningfulness and interestingness)	3 items	11-point discrete visual analogue scales (*Not helpful/ meaningful/ interesting at all* to *Very helpful/ meaningful/ interesting*)	*“How helpful did you find this session?”*	N/A
Acceptability: End of program satisfaction				
Overall program helpfulness	1 item	Four-point Likert item (*Not helpful* to *Very helpful*)	*“Overall, how helpful was the program?”*	N/A
Overall program satisfaction	1 item	Four-point Likert item (*Not at all satisfied* to *Very satisfied*)	*“Overall, how satisfied were you with the program?”*	N/A
Enjoyment of recreational activities	1 item	11-point scale (*Not enjoyable at all* to *Extremely enjoyable*	*“Overall, on a scale of 1 to 10, how much did you enjoy the recreational activities you participated in on camp?”*	N/A
Program recommendation to other bereaved young people	1 item	Yes/no question	*“Would you recommend the program to other young people who have a parent who has died from cancer?”*	N/A
Satisfaction questions (liked most, most useful, why recommend/not recommend, improvements suggestions)	4 items	Open ended	*“What did you like most about the program?”*	N/A
Satisfaction with facilitators (listening, supportiveness, understanding, respectfulness, creating a safe space, taking participant views/concerns seriously, knowledge, coping strategy provision)	8 items	Six-point Likert scale (*Strongly disagree* to *Strongly agree*)	*During the program, I felt that the facilitators… “listened to me*”; “*understood what I was going through*”	N/A
Appropriateness of program topics^*a*^ (perceived usefulness/ relevance/ practicability for day-to-day life in the months following GG attendance)	9 items	Five-point Likert scale (*Very unhelpful* to *Very helpful*)	*“There are ways to manage intense emotions that come from your grief”*; *“You share strengths and values with the person who died”*	N/A
Outcome measures				
Grief intensity – Texas Revised Inventory of Grief (TRIG; Present Feelings subscale) [49]	13 items	Five-point Likert scale (*Completely false* to *Completely true*)	“*I still cry when I think about the person who died*”	Present Feelings subscale scores showed sufficient internal consistency (*α* = .86) and split-half reliability (*r* = .88) [49].
Meaning-making – Grief and Meaning Reconstruction Inventory (GMRI; Personal Growth and Valuing Life subscales) [50]	11 items, 2 subscales (Personal Growth - one’s sense of having experienced positive change and increased resilience following the loss; Valuing Life - one’s sense of desire to live life to the fullest)	Five-point Likert scale (*Strongly disagree* to *Strongly agree*).	*“Since this loss, I’m a stronger person*”; “*I value and appreciate life more”*	The GMRI (overall and its subscales) has good convergent and discriminant validity [51]. Subscales demonstrated good internal consistency (α_Personal Growth_ = .83; α_Valuing Life_ = .76) [51].
Trauma coping abilities – Perceived Ability to Cope with Trauma Scale (PACT) [52]	20 items, 2 subscales (Trauma Focus - one’s ability to spend time processing the trauma; Forward Focus - one’s ability to move beyond the trauma)	Seven-point numeric rating scale (*Not at all able to* to *Extremely able to*	*“Look for the positive in things”*	The Coping Flexibility Scale shows good incremental, convergent and discriminant validity [52].
Unmet needs – Bereaved Cancer Needs Instrument (BCNI) [15]	57 items, 7 subscales (Help and Information About Grief, Time-Out From Grief, Planning For The Future, Support From Friends, Talking To Others With Similar Experiences, Dealing With Feelings, Family Connectedness)	Four-point Likert-type scale (*No need* to *Strong need*).	e.g., *“I currently need to be informed about grief and loss in a way that I can understand”*	Good convergent validity with a measure of psychological distress and high internal consistency for all subscales when validated in a sample of cancer-bereaved sibling and offspring AYAs [15].

#### Interviews

AYAs who consented to be interviewed participated in a 20-min semi-structured phone interview with a researcher which was recorded and transcribed for accuracy. Interview questions explored AYAs’ personal experience of the program in greater depth than surveys, with a focus on expectations of the program, skills and knowledge acquired, and areas for program improvement.

### Data handling and analysis

#### Missing data

Where more than 20% of a participant’s data was missing for a scale or score calculation, their data was excluded from that analysis. Where up to 20% of a participant’s data was missing for a scale or score, a prorated scale or score was calculated using the average of valid items.

#### Acceptability and appropriateness analyses

AYAs’ session engagement scores represent the average of their helpfulness, meaningfulness and interestingness scores for that session. It should be noted that attendees at some programs did not complete ratings for the Honouring Ceremony due to those sessions finishing late, or facilitators not wanting to disrupt the emotional flow of the session.

For AYAs’ ratings of overall program helpfulness and topic usefulness/relevance/practicability, the percentage endorsing the program/topic as helpful represents the combined number of *Helpful* and *Very helpful* responses, divided by the total number of responses. The same was done for overall program satisfaction (i.e., *Mostly satisfied* and *Very satisfied* responses).

AYAs’ responses to the open-ended satisfaction questions (what they liked most, what they found most useful, why they would/wouldn’t recommend the program, suggestions to improve the program) were categorised into key topics.

#### Outcome analyses

Measure totals were calculated. For the TRIG, responses are summed to produce an overall score (total TRIG), with higher scores representing greater grief intensity [49]. For the GMRI, scores are calculated for each subscale by summing its items, with higher scores representing a greater sense of meaning in that domain [50]. For the PACT, each subscale is scored by calculating the average rating of its items [52]. An overall Coping Flexibility Scale is calculated as the sum of the Trauma and Forward Focus subscales minus the polarity of these subscales. Higher Coping Flexibility scores represent a greater ability to employ coping strategies in both the Trauma and Forward Focus domains. For the BCNI, a score is calculated overall (total BCNI) and for each subscale by summing its items, with higher scores representing greater need in that domain [15].

To determine the effect of time on participants’ outcome scores, a one-way repeated measures multivariate analysis of variance (MANOVA) was conducted with time (T0, T2) as the independent variable and grief intensity (total TRIG), meaning-making (GMRI Personal Growth and Valuing Life subscales), trauma coping abilities (PACT Coping Flexibility Scale), and unmet needs (total BCNI) as dependent variables. For measures that had subscales and were found to be significant, further exploratory analysis was completed.

#### Interview analyses

A summary of participant interview responses was prepared under the key interview topics of program acceptability, meeting expectations, program benefits, personal growth, and improved coping strategies. Semantic coding of each response was completed and then codes were organised into categories (e.g., positive impression of program). The most common categories are presented under the key topic headings, with selected quotes as illustrative examples. One person conducted detailed coding of interviews, which was examined by another reviewer for accuracy.

## Results

### Participants

One hundred and nine bereaved AYAs (*M*_age_ = 16.63, range 12 to 25 years; out of 151 total attendees (72%) who attended a GG program participated in the program evaluation. The majority of AYA participants were female (68%), born in Australia (97%), and currently studying (82%). Seven percent of AYA participants identify as Aboriginal or Torres Strait Islander, and 6% reported they spoke a language other than English at home. Most AYA participants had experienced the death of a parent (*n* = 96, 88%). See Table 3 for details. Of the 109 total participants who provided data for at least one timepoint, 108 provided data at T0, 108 participants provided data at T1, 67 participants provided data at T2, and 13 participants took part in interviews (comparisons between participants who did and did not complete T2 found no differences for gender, age, or baseline BCNI or PACT scores). One participant completed T2 and session data without completing T0 or T1.

**Table 3:** Participant demographics (*n* = 109)

	*M*	*SD*
Age (years)	16.63	3.39
	*n*^*a*^	%
Gender		
Female	74	67.9
Male	35	32.1
Bereavement category		
Offspring	96	88.1
Sibling	13	11.9
Country of birth		
Australia	105	97.2
Canada	1	0.9
England	1	0.9
South Africa	1	0.9
Aboriginal or Torres Strait Islander	8	7.4
Speak language other than English at home	6	5.6
Studying	89	82.4
School	69	77.5
Technical College (TAFE)	4	4.5
University	16	18.0
Work		
Full-time	4	3.8
Part-time	8	7.6
Casual	39	37.1
Voluntary	2	1.9
Other work	3	2.9
Not employed	55	52.4

^*a*^*Minor discrepancies in missing data have resulted in some totals not reaching 109. It should be noted that AYAs were able to choose more than one work category (*e.g.*, if they were volunteering and working part-time)*

### Program acceptability and appropriateness

AYAs reported high levels of session engagement. Life Imprint was the highest scoring session (*n* = 120, *M* = 8.25, *SD* = 1.45), followed by Honouring Ceremony (*n* = 80, *M* = 8.23, *SD* = 1.94), Coping and Resources (*n* = 118, *M* = 8.20, *SD* = 1.64), Sharing Memories (*n* = 118, *M* = 7.92, *SD* = 1.61), and Introduction of Loved One (*n* = 121, *M* = 7.49, *SD* = 1.64). Understanding Grief was the lowest scoring session (*n* = 119, *M* = 7.40, *SD* = 1.84).

Reflecting on the overall program at its completion, 99% of participants agreed that GG was helpful or very helpful, and 99% reported they were mostly or very satisfied with the program. High enjoyment of recreational activities was also reported (*M* = 8.73, *SD* = 1.53). All but one AYA (99%) stated they would recommend the program to another young person whose parent or sibling has died from cancer. AYA ratings of satisfaction with facilitators ranged from 86 to 98% for all items, with high internal consistency (α > 0.8).

Of the 108 AYAs who completed the measures immediately post-program (T1), in the open-ended questions section when asked what they *most liked* about the program, the greatest number of AYAs (*n* = 33) mentioned the psychosocial sessions, with 14 specifically discussing the Honouring Ceremony session. This was followed by making new friends and meeting people (*n* = 29), recreation activities (*n* = 21), and the supportive and social environment of the program (*n* = 10).

When AYAs were asked about the *most useful* aspects of the program, the psychosocial sessions were mentioned most (*n* = 80). Of these, one-third discussed the Honouring Ceremony session, 12 specifically discussed the Coping and Resources session, and 11 mentioned the Life Imprint session. Following this, 11 AYAs mentioned being able to talk to and share their experiences with others.

When detailing *why they would recommend* the program, the most common responses were because it was helpful (*n* = 33), it provided an opportunity to meet other young people going through a similar experience (*n* = 26), they gained useful knowledge/skills (*n* = 22), and it was an enjoyable/positive experience (*n* = 13).

When asked about suggestions they have for *improving* the program, the greatest number of respondents (*n* = 50) mentioned making changes to the program structure. Of these AYAs, 20 reported wanting the program to be longer in duration and 19 reported wanting changes to be made to sessions (e.g., content, frequency, group size). This was followed by young people suggesting having more or different recreational activities (*n* = 16). Eighteen AYAs stated that they did not believe any changes should be made to the program.

Reflecting on the usefulness, relevance and practicability of the program topics for their day-to-day lives when asked as part of the three-month post-program survey, all program topics were perceived to be helpful by most young people (see Table 4).

**Table 4:** Program topics and the percentage of AYAs that rated the topic as being helpful (*n* = 66)

*Topic*	*Percentage of AYAs that rated the topic as helpful*
Other people are going through the same things as you are	92.42
You share strengths and values with the person who died	90.91
There are many different ways to grieve	87.88
There are ways to connect with positive memories of the person who died	86.15
There are ways to manage intense emotions that come from your grief	80.30
You are able to seek support when you need it	80.30
It is okay to talk about the person who died	80.00
There are ways to express your grief to yourself, your family and your friends	75.76
Mindfulness (e.g., being in the present moment)	61.54

### Program outcomes

The data was examined to ensure that assumptions were met for the MANOVA. The assumption of normality could not be met for the GMRI Valuing Life subscale due to a ceiling effect and so this variable was excluded from analyses. Removal of univariate and multivariate outliers identified visually using box plots and through computation of Mahalanobis Distances did not change the outcome of the main MANOVA and as such these cases were retained.

For the 63 participants who contributed sufficient data at T0 and T2 to be included in analyses, MANOVA revealed a statistically significant difference in AYAs’ outcome scores from T0 (baseline) to T2 (three months post-program), *F*(4, 59) = 6.73, *p* < .001, partial η^2^ = .31. Analysis of the individual dependent variables was conducted. To account for multiple comparisons, a Bonferroni correction was applied. Analysis revealed no difference in total TRIG from T0 to T2 (*F*(1, 62) = 3.14, *p* = .08, partial η^2^ = .05). However, the GMRI Personal Growth subscale (*F*(1, 62) = 6.73, *p* = .01, partial η^2^ = .10), PACT Coping Flexibility Scale (*F*(1, 62) = 10.26, *p* = .002, partial η^2^ = .14) and total BCNI (*F*(1, 62) = 16.64, *p* < .001, partial η^2^ = .21) were all significant at an adjusted alpha of 0.0125. Compared with T0, participants at T2 reported significantly greater personal growth-related meaning-making (*M*_*T0*_ = 25.97, *SD*_*T0*_ = 3.54; *M*_*T2*_ = 27.17, *SD*_*T2*_ = 3.81), trauma coping abilities (*M*_*T0*_ = 8.70, *SD*_*T0*_ = 1.56; *M*_*T2*_ = 9.31, *SD*_*T2*_ = 1.74), and lower unmet needs (*M*_*T0*_ = 143.91, *SD*_*T0*_ = 30.64; *M*_*T2*_ = 130.34, *SD*_*T2*_ = 35.82). These analyses were repeated with outliers removed. All significant results were retained with the exception of the GMRI Personal Growth subscale, which became non-significant (*p* = .04) at the Bonferroni adjusted alpha. To see whether our non-significant results could have occurred due to a lack of statistical power, we conducted power analyses using G*Power [53] with power (1 - β) set at 0.80 and α = .0125 (i.e. with Bonferroni adjustment), two-tailed. This showed us that sample sizes would have to increase to *N* = 409 for the TRIG and *N* = 107 for the GMRI Personal Growth subscale for T0 to T2 differences to reach statistical significance.

Exploratory analyses were conducted on each of the BCNI subscales in order to identify possible domains contributing to the total BCNI’s reduction from T0 to T2. As t-test normality assumptions were not met for many of the subscales, a series of non-parametric Wilcoxon Signed Rank Tests were performed. Results indicated that needs scores were significantly lower at T2 in comparison to T0 for all subscales except Family Connectedness (see Table 5).

**Table 5:** Median scores at T0, T2; BCNI subscale Wilcoxon Signed Rank Tests *z*-values, effect sizes (*r*)

*Subscale*	*n*	*Median*		*z*	*r*
		T0	T2		
Help and Information About Grief	64	14.00	12.00	−3.38***	−0.30
Time-Out from Grief	64	17.50	15.00	−2.86**	−0.25
Planning for The Future	64	16.00	14.00	−3.35***	−0.30
Support from Friends	64	17.00	15.50	−2.57*	−0.23
Talking to Others with Similar Experiences	64	15.00^a^	15.00a	−2.26*	−0.20
Dealing with Feelings	65	39.00	36.00	−3.13**	−0.27
Family Connectedness	65	29.00	26.00	−1.94	−0.17

^*a*^*It should be noted that despite the T0 and T2 medians being equal for this subscale, the Wilcoxon Signed Rank Test was significant. This is likely because this test is not calculated using the median, but rather is based on the sums of positive and negative ranks. For further clarity as to the direction of the reported change from T0 to T2, the means and standard deviations, and mean positive and negative ranks for this subscale are as follows – T0: M = 14.93, SD = 3.77; T2: M = 13.94, SD = 3.92; M*_*positive rank*_ *= 24.26; M*_*negative rank*_ *= 28.53*

* < .05, ** < .01, *** < .001

### Interviews

Key interview topics included program acceptability, meeting expectations, program benefits, personal growth, and improved coping strategies.

#### Program acceptability

The high satisfaction reported in surveys was also reflected in interviews, with the majority (*n* = 11, 85%) of interviewees reporting an overall positive impression of GG. In particular, the program’s structure and safe and welcoming environment was praised:

“*I think it was one of the best experiences that I've had, and I think that especially the structure that it went through was really a well thought out, and well designed, and applicable camp for the people that went… I really think it was an incredible experience*”.

Other interviewees described the program’s meaningfulness to them, and what they valued about it:

*“through acknowledging our loved one and letting us talk about who they are, what they mean to us, what they will always mean to you, and how they shaped who we are…even though that they're not here, we're able to use this space to honour, to just speak about how we feel, and to always remember* [them]*.”*

*“It’s an experience that will change your life, and will go through the sections of acknowledging, of reflecting, and honouring in a really thoughtful way. And you'll meet wonderful people who will be some of your greatest friends, and that you’ll have with you for, hopefully, the rest of your life.”*

#### Meeting expectations

Most interviewees (*n* = 11, 85%) reported that GG had met their expectations. Common expectations discussed were learning skills to express grief, meeting others with similar experiences, and fun and respite. The remaining two interviewees reported their expectations were only partially met, with reasons including being unable to make friends, feeling that there was not equal opportunity given for group members to share in the group space, or learning fewer coping skills than expected.

#### Program benefits

Interviews also reflected several program benefits that young people experienced during the program and in the months afterwards. Ten out of the thirteen AYAs interviewed (77%) talked about feeling less alone as a result of the program and identifying with other participants, with one AYA stating:

“*It was good to know people in my area that were also going through the same thing. Like having other people, younger people, I know I can connect with and you know, talk to about these things and not feel isolated as much*”.

Just over half talked about the benefit of psychoeducation about grief, with one young person saying “*They* [the sessions] *were really helpful for me just dealing with my grief and getting to know what grief is was really helpful”,* and another referring specifically to learning about the DPM:

“*We did this thing where we were talking about… the emotional side and then the productive, like, continue with your life side and so you're constantly moving between them. And that’s helped me realise that I could go in-between them when I needed to… So then that helped”.*

Eleven of thirteen interviewees (85%) identified making friends as a key benefit lasting well beyond the program, and nearly two-thirds reported having met others with similar experiences to their own who could understand what they were going through, with one AYA stating “*that’s something really huge that I gained from that camp, was the friends I made*” and “*everyone in there could relate to what I was going through*” and another explaining:

*“if you're trying to talk to your friends or someone* [else] *about it they don't understand how to explain and talk to you about things. They'll find it awkward and avoid it, but all these people* [at the GG program] *know where you're coming from”.*

Developing skills in expressing grief openly to others was also identified as a key benefit by just over half of the young people: “*if I’m struggling with something, I will tell the friends that I met* [on GG] *or I’ll let my dad know… I’m more accepting of the fact that I need to ask for help*”. Finally, two of thirteen interviewees mentioned feeling closer to their loved one as a result of the program, with one young person describing *“I wanted to feel closer to Mum and also, sort of understand how I was feeling…I think that’s the biggest thing that I took from the camp, is that I can still have a connection with Mum”*.

#### Personal growth

Interviews additionally reflected AYA participants’ awareness of personal growth following their loss, with eleven of thirteen interviewees (85%) citing that they felt different since their loved one’s death. AYAs reported this personal growth occurring in a number of areas, including maturation, gaining of new perspective and assuming more responsibility, with one AYA stating:

“*I think it's definitely made me a lot more stronger and a lot more aware of other people around me… it's just changed a lot of aspects of my life, really. How I treat other people. And how I expect to be treated by other people*”*.*

Another young person said:

“*I think there's a process of maturation that occurs, and I think that it makes you a more…- it makes you a wiser person than you necessarily would be for your age, and I think it makes you a more compassionate person. I think that it's… fast tracked the whole maturation process.”*

#### Improved coping strategies

Improvement of coping skills and abilities was also supported by interview data, with all interviewees mentioning various coping strategies they had learned from the program. These included talking about their emotions with others, accepting their grief and acknowledging the uniqueness of the individual grief experience, openness to facing grief rather than avoiding it, writing letters to their loved one and keeping their loved one’s memory alive. Illustrating this, one AYA stated that “*I’ve learnt to be able to cry and it’s okay to show your emotions”,* and others said:

*“I kind of used things that I have done with dad previously* [to cope]*. Whenever I contribute to an activity I kind of remember, it helps me get over it a little bit. Like whenever I go fishing or camping, like even the scent of the tents kind of remind me of him a little bit”.*

*“I think sometimes we can get a little bit lost through life and through just day to day things, but the camp really gave us a chance to sort of sit down with our feelings, and sort of go, ‘This is what's going on. This is how I feel. It's okay to feel this way’”.*

## Discussion

This paper provides evidence that GG was well received by young people bereaved due to cancer and that it significantly improved their psychosocial wellbeing. In particular, significant improvements were found in the core program outcomes, which had been identified as critical for preventing poor bereavement outcomes in this cohort. Our findings show that young people who attend the program experience the benefits that it was designed to deliver. It is expected that these benefits will provide AYAs with greater resilience and internal and external resources to walk through their grief experience and adjust to life following the death of their loved one.

### Acceptability and appropriateness

Young people were highly satisfied with the program, with strong convergence of survey and interview results. Almost all young people found the program helpful, satisfying and would recommend it to others. They also reported high enjoyment of recreational activities and satisfaction with facilitators. AYAs reported high engagement with all psychosocial sessions and for seven of the nine program topics at least 80% of young people reported them as helpful for their day-to-day lives. Regarding the helpfulness of the GG program’s topics, the examination of strengths and values shared with the deceased loved one, and the exploration of the shared grief experience between participants was the most highly endorsed topic. Supporting this finding, McDonald et al. [14] reported that support from other AYAs who have had a similar experience was the most highly endorsed unmet need domain for AYA siblings and offspring who have had a family member die from cancer. The next most endorsed GG topic in terms of its helpfulness was the examination of strengths and values shared with the deceased loved one. This topic is a major focus of the Life Imprint session - the session with the highest engagement score – which aims to facilitate meaning-making and strengthen AYAs’ continuing bonds with their loved ones. Examining shared strengths and values in this session may specifically strengthen AYAs’ internalised connections (an inner representation of the deceased person’s beliefs, values and personality) to their loved ones as a means of continuing bonds [44]. Previous research examining the Life Imprint session in detail also found it to be highly engaging and meaningful for AYAs, promoting reframing of life narratives in some participants [47], which can assist with coping and bereavement adjustment [33, 34]. Aligning with the existing literature, the present findings clearly highlight the importance of ensuring peer support provision is a core component of any AYA grief intervention.

### Outcomes

#### Trauma coping abilities

A significant increase in trauma coping abilities was observed from pre-program to the three-month post-program follow-up. This was further substantiated by AYA interviews conducted after the post-program follow-up, in which participants reported the learning and employment of a variety of coping strategies, with one AYA describing GG as being “*a very good opportunity to learn coping skills. And to learn more about why you feel the way you feel and grief as a whole*”. The PACT’s conceptualisation of coping as the flexible use of strategies relating to both facing the trauma experienced (e.g., engaging with painful emotions, remembering the event) *and* moving forward with one’s life (e.g., distraction from painful emotions, making plans, having fun) aligns with the DPM’s hypothesis of optimal adjustment to bereavement [28, 52]. As such, improvements seen from pre-program to follow-up suggest an increase in participants’ abilities to flexibly and adaptively engage in both loss- and restoration-oriented coping processes as a result of attending GG. Indeed, GG not only explicitly teaches AYAs the DPM’s core tenets, but also provides young people with a lived experience of it. In other words, at its core, the program’s format models and allows AYAs to practice oscillation between time that is spent focusing on grief, remembering the deceased, and engaging with the associated emotional and cognitive experiences, and time that is spent focusing on having fun with other young people and taking a break from grief.

#### Unmet needs

For all unmet need subscales other than Family Connectedness, significant reductions were observed from pre-program to three-month follow-up. The finding of no change in family connectedness needs was expected, as GG does not have a strong primary focus on improving family relationships and expressions of grief within the family unit. Reductions in most of the other areas of need were also as expected, as many of them represent the core aims and focus of GG – increasing knowledge about grief and support resources, facilitation of peer connections with other young people who have experienced the death of a family member due to cancer, facilitation of opportunities to express and learn about how to cope with grief-related emotions, and provision of respite and fun. Reductions in unmet needs associated with getting support from friends and practical assistance in planning for the future were also observed. It is possible that the opportunities provided on GG for AYAs to speak about their grief and loss experience enabled them to feel more comfortable talking to their friends about this once they returned home. There is also the possibility that at follow-up, AYAs’ new friendships with other AYAs they met on GG were meeting this need. Finally, whilst the BCNI’s Planning For The Future subscale reflects mostly practical needs (e.g., assistance with household chores, developing independence, budgeting), it is possible that GG assisted AYAs to know where and how to ask for help in these areas following the program. Interview data also reflected likely reductions in several of these unmet need areas. In particular, receiving of various forms of peer support were the most mentioned program benefits.

#### Meaning-making

Interviews with AYAs demonstrated awareness of several areas of personal growth following the death of their loved one. It is possible that this resulted from the opportunity provided on GG for AYA participants to reflect on meaning made as a result of loss, and therefore to further integrate the loss into their self-narrative [30, 31, 33]. Furthermore, given the power analysis findings, we cannot confidently rule out a possible impact of low power on the lack of significant change in personal growth-related meaning making.

#### Grief symptoms

No change was observed in grief intensity, with post-hoc power analysis suggesting that this finding is unlikely to be due to insufficient power. Whilst we do not know with certainty why there was no change, it may have been that some young people had avoided engaging with grief prior to the program, and so the program may have provided a space for them to engage with their grief, potentially increasing or sustaining their grief intensity scores in the months following GG. Illustrating this possibility, in an interview, one AYA reported,

“*I was always pushing* [grief] *back and I needed a set time or a set thing telling me what to do… I think, for me, it was just that I actually did get to spend that time reflecting… Which I, in all honesty, I hadn’t really done*”.

Another possibility is that perhaps no change in these symptoms should be expected. On reflection, as this is a measure of grief symptoms that for the most part constitute a normal and even healthy response to loss [54], then perhaps we would expect these to persist for some time, especially within the timeframe this evaluation was conducted, regardless of whether an intervention is administered or not.

### Limitations and future research

Without a control group, we were unable to account for potential changes in outcomes over time due to natural growth and recovery processes unrelated to the intervention, or due to participants potentially receiving other forms of support from Canteen. Additionally, whilst McDonald et al. [14] found that the overall needs of AYA siblings and offspring bereaved due to cancer are very similar, subtle differences were evident. However, as the majority of AYAs in the present study were bereaved offspring with only a small number of bereaved siblings participating, it was not possible to assess any differences in how the program may have impacted the two groups. In addition, although personal growth and meaning-making were described in interviews, given the small number of interviewees, it is unclear if this meaning-making occurred equally for all AYA participants. In this way, the potential of bias being present in the small subset of AYAs who agreed to be interviewed must be considered. For instance, it may be that interviewees tended to have had a more significant meaning-making experience on GG or were better able to recognise and articulate personal growth than the majority of attendees. Finally, given Family Connectedness was the only BCNI subscale that did not show significant reduction from pre-program to follow-up, future research could consider including family in the intervention (e.g., [21]) to improve familial relationships and support.

### Clinical and policy implications

GG is a sustainable community-based program to which healthcare providers can refer young people who have experienced the death of a family member to cancer. This is especially important given these young people’s unmet needs and that the provision of such services is generally out of scope for primary, secondary and tertiary healthcare. Reflecting GG’s long-term sustainability, since its initial piloting, GG has been run 10 times, and has informed the development of an online grief program alternative for AYAs who cannot attend GG in person. This has been especially important during travel and social distancing restrictions associated with COVID-19. Furthermore, GG offers AYAs therapeutic opportunities beyond those that can be provided through typical one-to-one counselling services – namely peer support and the lived experience of the DPM.

Healthcare services and hospitals may be unaware that their patients have adolescent or young adult children, so when providing end-of-life care to cancer patients, they should implement systems to assist with the identification of AYA family members who may benefit from referral to community-based support programs like GG (e.g., [55]) either as a standalone program or in conjunction with other psychological support. Additionally, greater awareness within the community of both the needs of bereaved young people and how and why they should access support, will assist them to access the support they need.

## Conclusions

Given the indelible impact that a parent or sibling’s death to cancer can have on young people, it is critical that theoretically driven and evidence-based programs addressing this impact are available and accessible to bereaved young people. The current study provides strong evidence for the acceptability, appropriateness and effectiveness of the GG program in meeting this need and demonstrates the vital role community organizations have in the provision of such support.

## Data Availability

The data that support the findings of this study are not publicly available as they contain potentially identifying and sensitive information. No ethical approval was obtained for the sharing of this dataset with researchers external to this project. Contact the corresponding author (Pandora Patterson) for further information.

## Abbreviations

AYA: adolescent and young adult
GG: Good Grief
DPM: dual process model
TRIG: Texas Revised Inventory of Grief
GMRI: Grief and Meaning Reconstruction Inventory
PACT: Perceived Ability to Cope with Trauma Scale
BCNI: Bereaved Cancer Needs Instrument
